# Study of the obstructive sleep apnea syndrome in cerebral infarction patients

**DOI:** 10.3389/fneur.2023.1132014

**Published:** 2023-06-21

**Authors:** Tien Hoang-Anh, Quy Duong-Minh, Nhi Nguyen-Thi-Y, Sy Duong-Quy

**Affiliations:** ^1^Cardiology Department of University of Medicine and Pharmacy, Hue University, Hue, Vietnam; ^2^Sleep Lab Center, Lam Dong Medical College and Bio-Medical Research Center, Dalat, Vietnam; ^3^Immuno-Allergology Division, Hershey Medical Center, Penn State Medical College, Hershey, PA, United States; ^4^Department of Outpatient Expert Consultation, Pham Ngoc Thach University of Medicine, Ho Chi Minh City, Vietnam

**Keywords:** obstructive sleep apnea syndrome (OSAS), OSA, apnea-hypopnea index (AHI), stroke, respiratory polygraphy

## Abstract

**Introduction:**

Obstructive Sleep Apnea Syndrome (OSAS) is the most common respiratory disorder during sleep. Many studies have shown an association between obstructive sleep apnea syndrome and stroke, and OSAS has not been adequately considered in Vietnam compared to the actual clinical dangers. This study aims to assess the prevalence and general characteristics of obstructive sleep apnea syndrome in patients with cerebral infarction and investigate the relationship between obstructive sleep apnea syndrome and the severity of cerebral infarction.

**Methods:**

Descriptive cross-sectional study. We identified 56 participants from August 2018 to July 2019. Subacute infarcts were identified by neuroradiologists. For each participant, vascular risk factors, medications, clinical symptoms, and neurological examination were abstracted from the medical record. Patients were taken for history and clinical examination. The patients were divided into two groups according to their AHI (Apnea-Hypopnea Index) (<5 and ≥5).

**Results:**

A total of 56 patients were registered for the study. The mean age is 67.70 ± 11.07. The proportion of men is 53.6%. AHI has a positive correlation with neck circumference (*r* = 0.4), BMI (*r* = 0.38), the Epworth Sleepiness Scale (*r* = 0.61), LDL cholesterol (*r* = 0.38), the Modified Rankin Scale (*r* = 0.49), NIHSS (National Institutes of Health Stroke Scale) (*r* = 0.53), and an inverse correlation with SpO_2_ (*r* = 0.61).

**Conclusion:**

Obstructive sleep apnea Syndrome is a factor in the prognosis of cerebral infarction as well as cardiovascular diseases such as hypertension. Thus, understanding the risk of stroke in people with sleep apnea is necessary and working with a doctor to diagnose and treat sleep apnea is important.

## Introduction

Obstructive sleep apnea (OSA) is a chronic sleep-related breathing disorder characterized by recurrent partial or complete cessation of airflow due to upper airway obstruction during sleep that results in sleep fragmentation, intermittent hypoxia, and hypercapnia leading to increased sympathetic nervous system activity (1). OSA has a prevalence reaching 8.5% of the adult population in Viet Nam considering all OSA severities (2). Night-to-night AHI variability does not have a definitive explanation but is reported in polysomnography measurements and should be considered in treatment decisions (3, 4).

Patients with OSA may exhibit loud and chronic snoring, gasping episodes during sleep, sleepiness, obesity, and increased neck circumference (5). OSA is associated with physical examination alterations and systemic complaints, including daytime fatigue and impaired concentration (6). OSA is also an independent risk factor for arterial hypertension, stroke, ischemic heart disease, cardiac arrhythmia, and heart failure (1).

Cerebral infarction tends to increase because the risk factors for the disease do not decrease such as a sedentary lifestyle, a high-fat diet such as fast food, high-sugar drinks, stress and high blood pressure, diabetes, and smoking become common (7).

Patients with ischemic stroke often develop sleep apnea and are common within the first 24 h. Disorderly breathing can be worse if the stroke happens while patients are asleep. OSA is the most common kind of sleep apnea that takes place after a stroke. OSA affects up to 70% of people with a stroke, compared to 30% of the overall population. A major aspect of medical care is the detection and treatment of OSA, as a treatment for OSA may enhance a person's recovery and decrease the risk of additional strokes. The relationship between stroke and OSA is bidirectional:

Kim et al. studied 80 patients with stroke and concluded that OSAS could cause dysfunction in patients with cerebrovascular accidents (8).

Ahn et al., when studying 293 patients with 159 men and 134 women with acute cerebral infarction, 63.1% (111 men, 74 women) had SAS, mainly OSAS with AHI >10 and at the same time concluded the relationship between SAS and the score of the National Institutes Of Health Stroke Scale (NIHSS: National Institutes Of Health Stroke Scale) and the adjusted Rankin Scale (mRankin: Modified Rankin Scale) high and worse long-term outcomes compared with the group without SAS (9).

Mattaliano et al. when studying 130 patients with acute cerebral infarction, the results showed that 61.9% had OSAS, most of which were men accounting for 67.1%. This study confirms the high prevalence of OSA in stroke patients and shows an association between OSA and target organ damage (10).

Researchers have identified OSA as an independent risk factor for stroke. This means that people with OSA are at higher risk of stroke, even if there are no other risk factors. There are several possible reasons for why people with OSA have a higher risk of stroke. Repeated collapses of the airway during sleep create negative air pressure within the chest, which may slow the flow of blood to the brain, thereby altering cerebral vascular abnormalities. OSA increases the risk of developing heart disease, hypertension, diabetes, and heart arrhythmias. These and other health consequences of OSA can increase the risk of stroke. These and other effects may be associated with an increased risk of stroke.

Many studies have shown an association between obstructive sleep apnea syndrome and hypertension, coronary artery disease, arrhythmia, and systolic heart failure (11, 12). Numerous studies have also mentioned the association between sleep apnea syndrome and stroke (13, 14). Based on this situation, we conducted a study that aims to achieve two objectives:

1. Evaluation of prevalence and general characteristics of obstructive sleep apnea syndrome in patients with cerebral infarction.
2. Investigate the relationship between obstructive sleep apnea syndrome and the severity of cerebral infarction.

## Methods

### Subjects

A total of 56 patients were diagnosed with subacute cerebral infarction and treated at the Cardiology Department of Hue University of Medicine and Pharmacy Hospital.

Obstructive sleep apnea Diagnostic Criteria were recommended by the American Academy of Sleep Medicine 2008 (AASM: American Academy of Sleep Medicine) (15). The patient suspected of OSAS must fulfill criterion A or B, plus criterion C. These are as follows:

A. Excessive daytime sleepiness that is not better explained by other factors.
B. Two or more of the following that are not better explained by other factors:

- Choking or gasping during sleep
- Recurrent awakenings from sleep
- Unrefreshing sleep
- Daytime fatigue
- Impaired concentration

C. Overnight monitoring demonstrates five or more obstructed breathing events per hour during sleep. These events may include any combination of obstructive apneas/hypopneas or respiratory effort–related arousals, as defined below.

#### Inclusion criteria

All subjects aged over 15 and fulfilling the criteria for a clinician-confirmed diagnosis of subacute cerebral infarction as defined by neuroradiologists were included in the study.

#### Exclusion criteria

- Patients with acute and severe diseases, chronic obstructive pulmonary disease or chronic respiratory failure, and cerebral diseases such as cerebral tumors, meningitis, and encephalitis.
- Patients who do not agree or cannot participate in the study.
- The patient is taking drugs that affect respiratory polygraph.

### Methods

We identified 56 participants from August 2018 to July 2019. Subacute infarcts were determined by neuroradiologists. For each participant, vascular risk factors, medications, clinical symptoms, NIHSS, Epworth, and neurological examination were abstracted from the medical record. The study sample included 40 subacute cerebral infarction patients with OSA and 16 subacute cerebral infarction patients without OSA. Assess outcomes at 3 months after stroke with mRankin, face-to-face visit, or telephone.

#### Study design

It was a descriptive cross-sectional research method with follow-up.

#### Anthropometry

Patients were weighed using a calibrated scale to the nearest 0.1 kg, and height (to 0.1 cm) was measured with a stadiometer (Medisol, Vietnam). Body mass index (BMI) was classified by WHO in 1986.

#### Respiratory polygraphy

The respiratory polygraphy system used in the study is the Embletta GOLD. Embletta GOLD recorded nasal airflow, snoring using a nasal pressure cannula, blood oxygen saturation, heart rate by pulse oximetry, and respiratory effort using a thoracic piezoelectric chest belt.

The device will be provided to patients with an explanation of how to use it in advance, as well as precise instructions on the correct positioning of the equipment's sensors and monitoring. Patients will also conduct several tests to familiarize themselves with the instrument's operating instructions. When the device is returned the next day, raw data files will be uploaded to a computer and recorded automatically and manually by trained physicians from the Study Group. A respiratory polygraphy recording will be deemed valid if the recording duration is ≥5 h. Sections with artifacts or poor signals will be excluded from the analysis. If respiratory polygraphy is not valid, it will be repeated within the next 7 days.

#### OSA criteria

The diagnostic criteria for adult OSA as defined by the American Academy of Sleep Medicine (16).

Overnight monitoring demonstrates five or more obstructed breathing events per hour during sleep. These events may include any combination of obstructive apneas/hypopneas or respiratory effort–related arousals, as defined below.

This report also proposed a grading of severity of OSAS based on the frequency of abnormal respiratory events during sleep: Mild: ≥5 but <15 events/hour of sleep; Moderate: 15–30 events/hour of sleep; Severe: More than 30 events/hour of sleep.

#### The definition and severity of subacute infarction

Classification of cerebral infarction by time (17):

- Acute cerebral infarction: from the first day of the 1st week after symptom onset.
- Subacute cerebral infarction: from the second week to 1 month.
- Chronic cerebral infarction: After 1 month.

The severity of cerebral infarction was based on NIHSS and mRankin. Stroke severity was categorized as follows: no stroke symptoms (0), minor stroke (1–4), moderate stroke (5–15), moderate to severe stroke (16–20), and severe stroke (21–42). In our study, the highest score was 14, thus, we divided it into two groups: 0–4 and 5–14 (18).

The original mRankin defined grade 1 as “No significant disability: able to carry out all usual duties,” and defined grade 2 as “Slight disability: unable to carry out some of the previous activities.” Patients with an mRs score ≤ 2 by definition are independent (19).

#### Data collection

All data on age, gender, height, weight, BMI, medical and family history, clinical characteristics, and respiratory polygraphy parameters (AHI, SpO_2_, pulse, and frequency of snoring) of the study subjects were collected for statistical analyses.

#### Ethical approval

All procedures performed in studies involving human participants followed the institutional and/or national research committee's ethical standards and the 1964 Helsinki Declaration and its later amendments or comparable ethical standards. The study was approved by the Hue University of Medicine and Pharmacy Institutional Ethical Review Board. Informed consent was obtained from all individual participants included in the study.

#### Statistical analysis

SPSS 22.0 software (IBM Corporation, Armonk, NY, USA) was used to analyze these collected data. Qualitative data are expressed as percentages or rates and compared with the Chi-squared test. Continuous variables were presented as mean ± standard deviation (SD) and compared with a *t*-test between 2 groups and a one-way analysis of variance among groups, followed by paired comparison with the least-significant difference test. A value of *p* < 0 0.05 was considered statistically significant.

## Results

### General characteristics of study subjects

During the study period, 56 patients with subacute infarcts met the inclusion criteria and were enrolled in this study. The demographic characteristics (gender, age, neck circumference, waist circumference, and BMI) of the study population are shown in Table 1. There was no statistically significant difference between age and gender for both groups. The mean age is 67.70 ± 11.07. The proportion of men is 53.6% (Table 1).

**Table 1:** Age group, gender, body measurements, and cardiovascular disease risk factors.

**General characteristics (*****n*** = **56)**		**Non-OSAS**		**OSAS**		**Total**		***p***
	***n***	**%**	***n***	**%**	***n***	**%**	
Gender	Male	7	12.5	23	41.1	30	53.6	>0.05
	Female	9	16.1	17	30.4	26	46.4	
Age	< 50	1	1.8	1	1.8	2	3.6	>0.05
	50–69	9	16.1	24	42.9	33	58.9	
	≥70	6	10.7	15	26.8	21	37.5	
Body measurements	Neck circumference (cm)	36.19 ± 1.07		37.25 ± 1.92		36.95 ± 1.24		**< 0.05**
	Waist circumference (cm)	84.25 ± 2.05		84.95 ± 1.87		84.75 ± 1.93		>0.05
	BMI (kg/m^2^)	19.09 ± 2.37		21.56 ± 2.98		20.86 ± 3.02		**< 0.05**
Smoking history		5	8.9	16	28.6	21		>0.05
Overweight and obesity		1	1.8	13	23.2	14		**< 0.05**
Hypertension		13	23.2	38	67.9	51		>0.05
Dyslipidemia		9	16.1	30	53.6	39		>0.05

BMI, body mass index; OSAS, Obstructive Sleep Apnea Syndrome. The bold values mean statistically significant difference (with *p* < 0.05).

The OSAS group was statistically higher in neck circumference and BMI (Body Mass Index) than the group without OSAS (*p* < 0.05).

Cardiovascular risk factors include smoking history, overweight and obesity, hypertension, and dyslipidemia. The results of this study noted that the prevalence of overweight and obesity was statistically higher in the group with OSAS (*p* < 0.05).

### Clinical and laboratory features

Table 2 summarizes the severity of OSA based on AHI results measured from respiratory polygraphs. According to our research, the moderate OSA group accounted for the highest rate of 75%; the mild and severe OSA groups accounted for the same with 12.5% (Table 2).

**Table 2:** The classification of OSA severity.

**AHI (event/hour)**	***n***	**Ratio (%)**
5 to < 15	5	12.5
15–30	30	75.0
>30	5	12.5

AHI, apnea-hypopnea index.

The results table below shows the index for the respiratory polygraph and the characteristics of blood pressure which include average SpO_2_, Lowest SpO_2_, measurement time, systolic blood pressure, and diastolic blood pressure. The average and lowest SpO_2_ were statistically smaller in the OSA group than in the non-OSA group (*p* < 0.05). We are interested in systolic blood pressure and diastolic blood pressure. In addition, we found that diastolic blood pressure in the OSAS group was statistically higher than in the non-OSAS group (*p* < 0.05) (Table 3).

**Table 3:** Index for the respiratory polygraph and characteristics of blood pressure.

**Characteristics**		**Non-OSAS**	**OSAS**	***P***
Average SpO_2_ (%)	Median	95.8	90.0	**< 0.05**
	Variation	95–97	85–95.2	
Lowest SpO_2_ (%)	Median	93.5	83	**< 0.05**
	Variation	88–95	79–92	
Measurement time (minutes)	$\bar{X}$ ± SD	500.1 ± 28.8	483.2 ± 35.7	>0.05
Systolic blood pressure (mmHg)	Median	150	150	>0.05
	Variation	110–180	120–260	
Diastolic blood pressure (mmHg)	Median	80	85	**< 0.05**
	Variation	70–100	60–120	

The bold values mean statistically significant difference (with *p* < 0.05).

**Figure 1:**
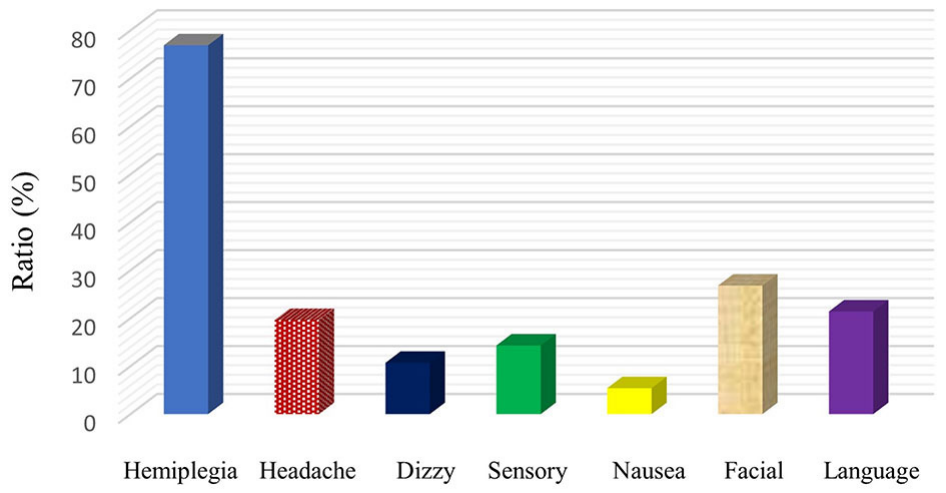
Clinical symptoms on admission.

Among the symptoms of patients with cerebral infarction, hemiplegia was the highest at 76.8%. Followed by facial paralysis, aphasia, and headache with respect ratio is 26.8, 21.4, and 19.6%. In addition, patients may have dizzy (10.7%), sensory disturbances (14.3%), and nausea (5.4%) (Figure 1).

Table 4 describes the characteristics of NIHSS and mRankin of study groups (compare between OSAS and non-OSAS groups). NIHSS in the OSAS group was statically higher than in the non-OSAS group (The median NIHSS for each group was 5 vs. 2) (*p* < 0.05) Similarly, the mRankin score in the OSAS group was also statistically higher than in the non-OSAS group (48.21 and 23.21% vs. 26.79 and 1.79%) (*p* < 0.05) (Table 4).

**Table 4:** Characteristics of NIHSS.

		**Non-OSAS**		**OSAS**		***p***
		***n***	**%**	***n***	**%**	
NIHSS	0–4	15	26.8	18	32.1	**< 0.05**
	5–14	1	1.8	22	39.3	
	Median	2		5		**< 0.05**
	Variation	0–5		1–12		
mRankin score	0–2	15	26.79	27	48.21	**< 0.05**
	>2	1	1.79	13	23.21	
	Trung bình	0.44 ± 0.81		1.98 ± 1.12		**< 0.05**

NIHSS, National Institutes of Health Stroke Scale. The bold values mean statistically significant difference (with *p* < 0.05).

**Figure 2:**
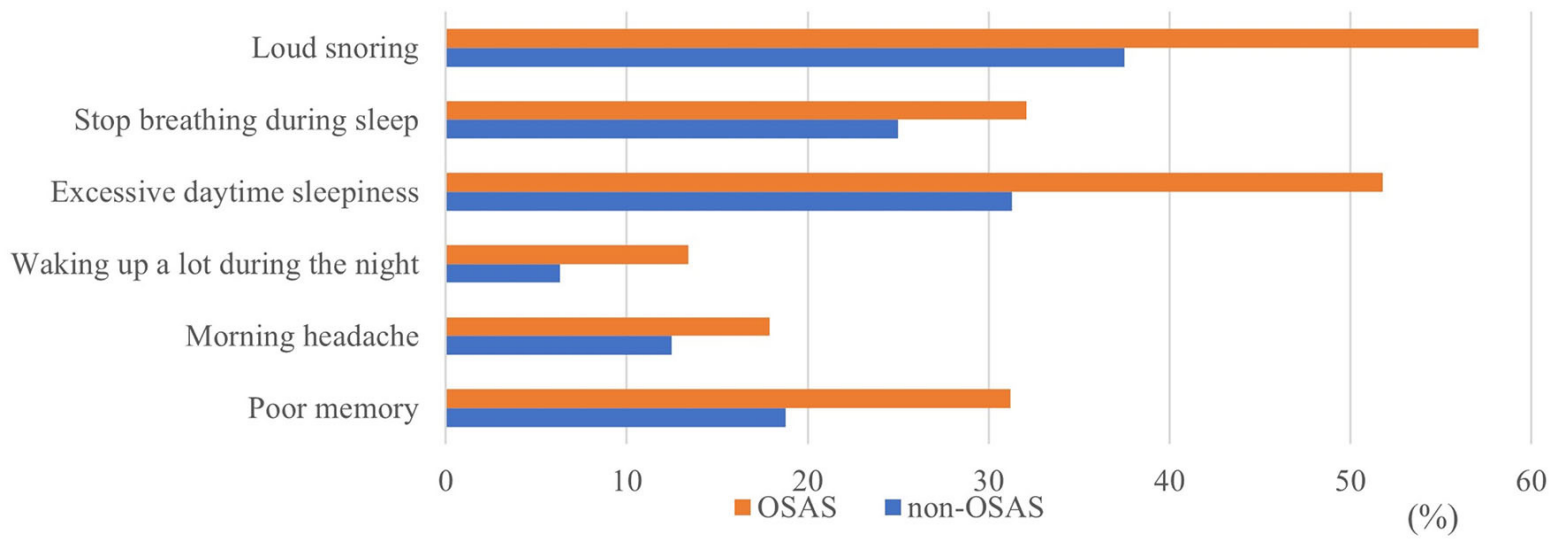
Symptoms of obstructive sleep apnea.

Figure 2 summarizes the clinical symptoms of obstructive sleep apnea including loud snoring, non-breathing during sleep, excessive daytime sleepiness, waking up a lot during the night, morning headache, and poor memory. Loud snoring during sleep accounted for the highest rate (38/56 patients); ~80% of patients in the OSAS group have loud snoring.

### Correlation between AHI and neck circumference, BMI, LDL cholesterol, NIHSS score, mRankin

**Figure 3:**
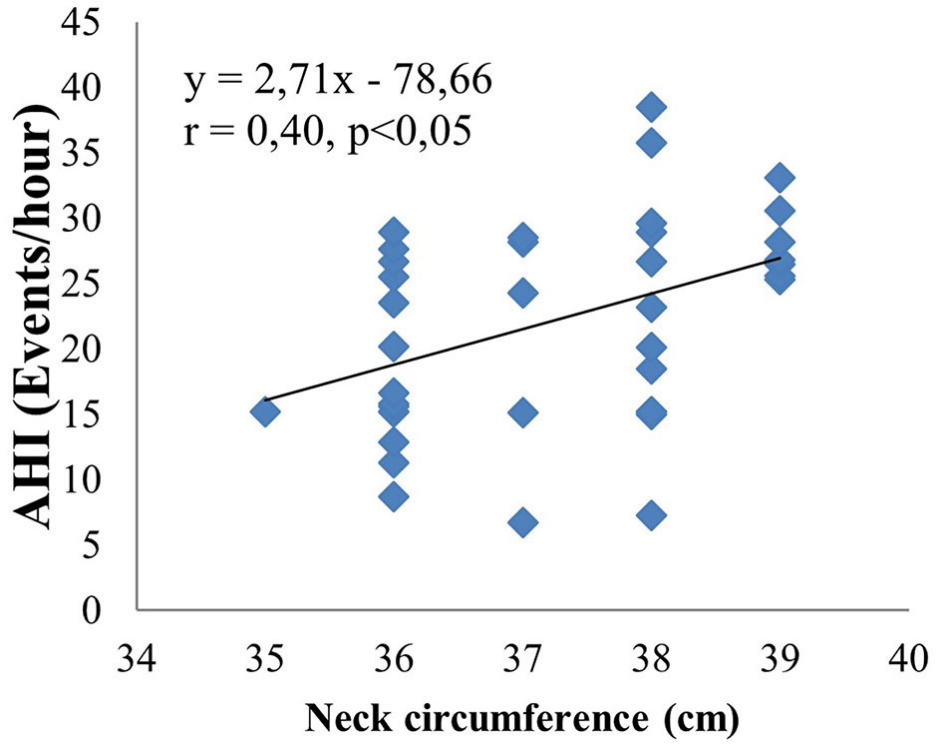
Correlation between AHI and neck circumference.

**Figure 4:**
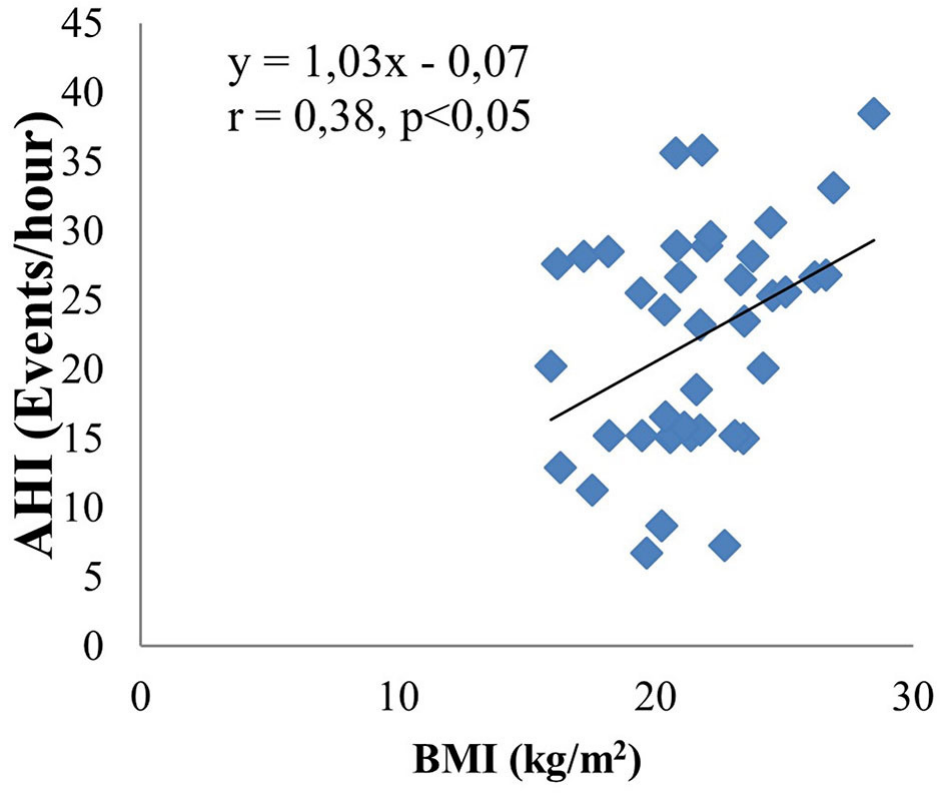
Correlation between AHI and BMI.

**Figure 5:**
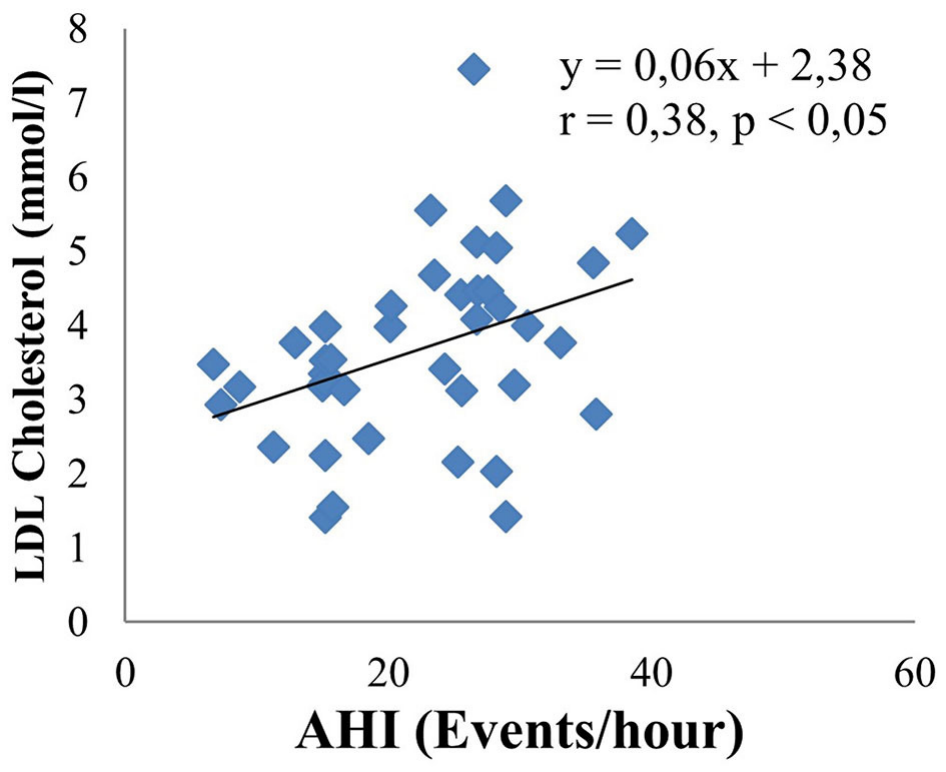
Correlation between AHI and LDL Cholesterol.

**Figure 6:**
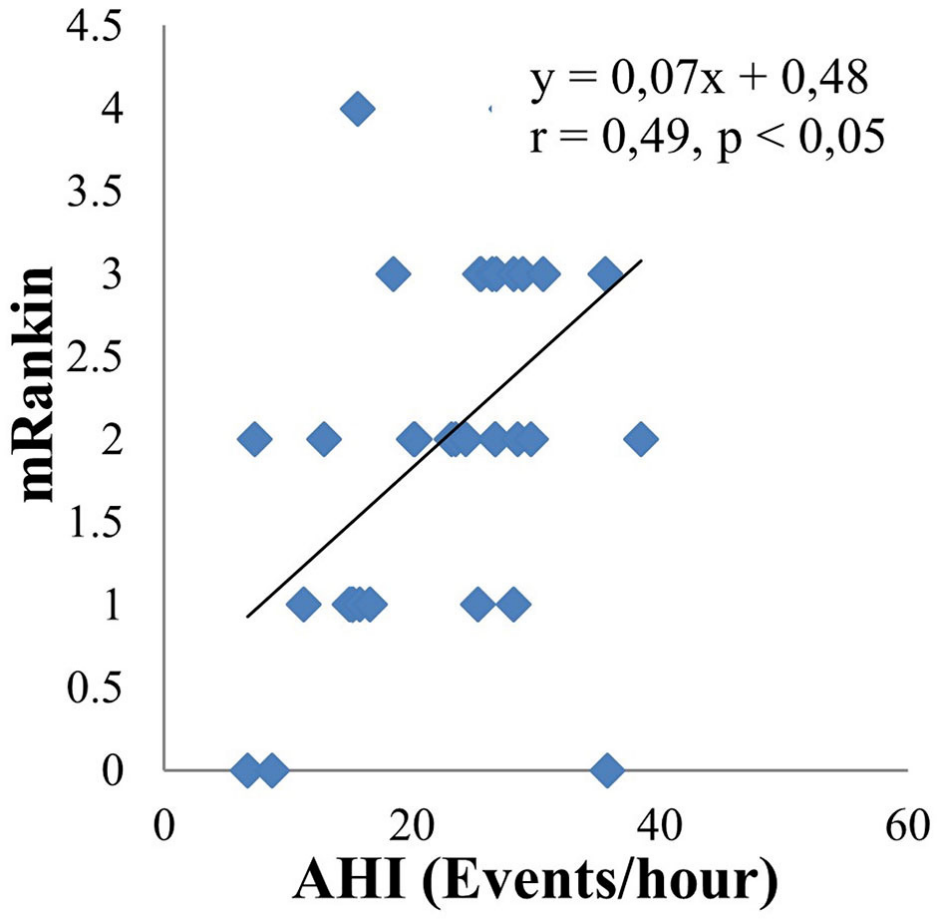
Correlation between AHI and mRankin.

**Figure 7:**
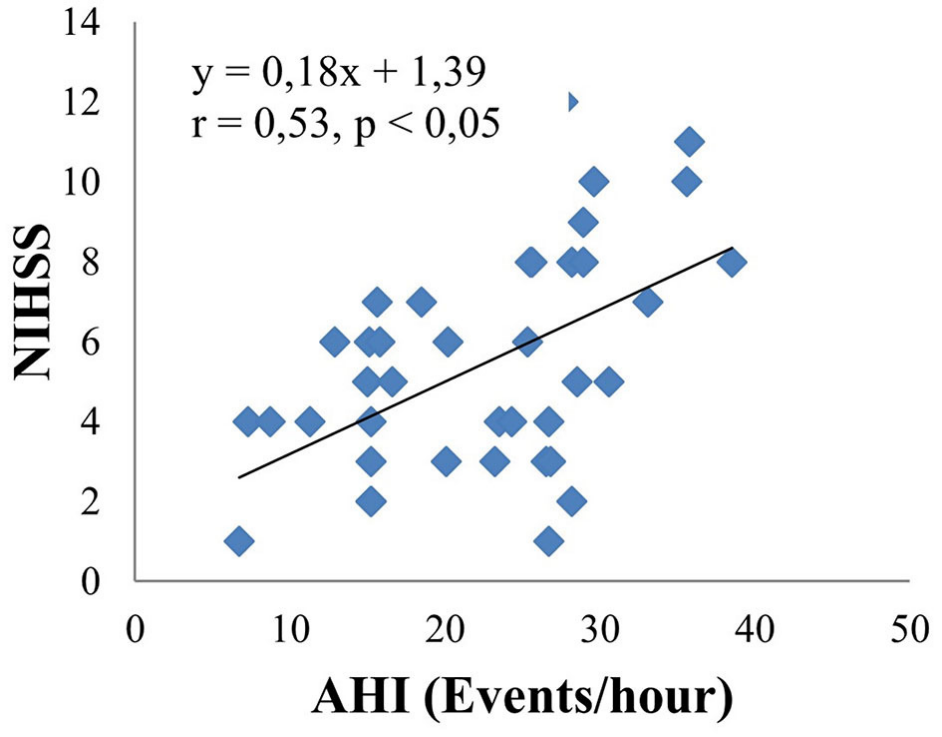
Correlation between AHI and NIHSS.

The main objective of this study is to investigate the relationship between obstructive sleep apnea syndrome and the severity of cerebral infarction. Therefore, we analyzed in relation to risk factors such as neck circumference, BMI, LDL cholesterol, mRankin, and NIHSS. The results are shown by linear equations. There is a moderate positive correlation between AHI and neck circumference (*r* = 0.4, *p* < 0.05) (Figure 3). There are similar results between AHI and BMI (*r* = 0.38, *p* < 0.05) (Figure 4); AHI and LDL Cholesterol (*r* = 0.38, *p* < 0.05) (Figure 5); AHI and mRankin (*r* = 0.49, *p* < 0.05) (Figure 6). Especially, we find a strong positive correlation between AHI and NIHSS (*r* = 0.53, *p* < 0.05) (Figure 7).

## Discussion

### General and clinical characteristics

Regarding the general characteristics of the study subjects, this study was conducted on 56 patients with cerebral infarction with an average age of 67.70 ± 11.07. The study did not show a statistically significant age difference between the OSAS and non-OSAS groups. Age is also one of the critical risk factors for OSAS. The prevalence of OSA increases with age among adults and tends to stabilize by age 65 (20). In the OSAS group, men account for 53.6%. This result is consistent with several studies, such as the study of Asha'ari Zamzil Amin with a male-female ratio of 80/38, and author Sy Duong Quy gave the result that this ratio is 1.2/1 (2, 21). This result has been documented in the literature as the prevalence of OSA is higher in men than in women, and most population-based studies show that the prevalence of OSA is 2 to 3 times higher in men (22, 23). In fact, women often do not present with the classic symptoms of OSA (loud snoring, sleep apnea, and excessive sleepiness). They mainly complain of a lack of energy and fatigue. Sex hormones may have an important role in the pathogenesis of OSA. Evidence is that OSA is more common in post-menopausal women than in premenopausal women, and hormone replacement therapy for post-menopausal women may protect them against the disorder (24).

Our study has the result that BMI in the OSAS group is 21.56 ± 2.98 (kg/m^2^), statistically higher than the non-OSAS group [19.09 ± 2.37 (kg/m^2^) (*p* < 0.05)]. This result is similar to Duong Quy Sy's study with the mean BMI in the group with OSAS of 23.85 ± 3.43 (kg/m^2^), which is statistically significantly higher than in the non-OSAS group with 21.58 ± 2.98 (kg/m^2^) (*p* < 0.05) (2). This result shows the relation between BMI and OSAS, which is also consistent with the research results of Carmine F. et al. by finding a moderate positive correlation between BMI and AHI (*r* = 0.33, *p* < 0.001) (25). Overweight and obesity increase the severity of OSAS because fat accumulation in some areas, especially around the upper respiratory tract, easily leads to the risk of fat deposition near the pharynx causing narrowing of the upper airway; changes in neural compensatory mechanisms to maintain airway openness; the respiratory control system is unstable. In our study, the rate of overweight and obesity was 25% (23.2% in the OSAS group and 1.8% in the non-OSAS, *p* < 0.05).

In our study, the neck circumference in the OSAS group was 37.25 ± 1.92 (cm) higher than in the non-OSAS group. This result can be explained because neck circumference is one of the manifestations of upper body fat, which is one of the important factors of OSAS. Therefore, neck circumference is a better predictor of OSAS than waist circumference and other metabolic syndrome factors. However, the direct role of neck circumference in the pathogenesis of OSAS has not been clearly defined (26).

The lowest median SpO_2_ in the OSAS group was statistically significantly lower than that in the non-OSAS group (*p* < 0.05). The background SpO_2_ in the OSAS group was also significantly lower than that of the non-OSAS group (*p* < 0.05). The results are quite similar to a few other studies around the world. Following Mattaliano et al., the background SpO_2_ in the OSAS group (+) was 92.7 ± 2.9, which was statistically significantly lower than the OSAS group (–) was 94.0 ± 2.2. Meanwhile, the lowest SpO_2_ in the OSAS group (+) was 81.9 ± 7.6, which was statistically significant compared with the OSAS group (–) was 87.7 ± 4.3 (*p* < 0.01) (10).

Snoring is one of the symptoms of OSAS and has the highest prevalence in this study. It is a sound produced by the vibration of the upper respiratory tract soft tissues during sleep. A 14-year longitudinal study found that 13% of adults snore. Factors associated with snoring include male gender, obesity, smoking, and asthma. In addition, snoring is strongly associated with increased all-cause mortality (27). The study's results also have shown that excessive daytime sleepiness is higher in the OSAS group than in the non-OSAS group. This result is an important and common symptom of OSAS. Daytime sleepiness can mean losing alertness or falling asleep under inappropriate circumstances. People are considered excessively sleepy when they are not alert enough to perform the tasks of daily living.

### Relationship between obstructive sleep apnea syndrome and cerebral infarction

In this study, diastolic blood pressure in the OSAS group was statistically higher than in the non-OSAS group. The study of Chen et al. in the chronic cerebral infarction group showed similar results that there was a statistical difference in hypertension between the group with OSAS and the control group (28). This result can be explained by the fact that OSAS patients with apnea sleep lead to increased sympathetic activities and endothelial dysfunction, ultimately resulting in vascular structural modifications, vasoconstriction, cardiovascular events, and hypertension (14).

The NIHSS and mRankin scores were statistically higher in the OSAS group than in the non-OSAS group. The study of Ahn et al. showed that the mRankin score in the OSAS group (1.68 ± 1.89) was statistically higher than in the non-OSAS group (1.18 ± 1.65) and this result is quite similar to our study.

Obstructive sleep apnea Syndrome is independently associated with hypertension, insulin resistance, impaired glucose tolerance, and dyslipidemia. Our results show that the concentration of total cholesterol, LDL-C in the OSAS group was statistically significantly higher than that in the non-OSAS group. The remaining indexes such as fasting intravenous glucose, HDL cholesterol, and triglyceride had no statistically significant differences between the two groups. There is a statistically significant mean positive correlation between LDL-C and AHI with the regression equation: y = 0.06x + 2.38; r = 0.38; *p* < 0.05. Analyzing the multivariate correlation between AHI and other factors, we found that if AHI increased by 1 event/hour, the NIHSS increased by 0.23 points. In contrast, when LDL cholesterol increased by 1 mmol/l, the NIHSS decreased by 0.78 points. In addition, the study also showed a strong positive correlation between AHI and NIHSS (*r* = 0.53, *p* < 0.05), as well as a moderate positive correlation between AHI and mRankin score (*r* = 0.49, *p* < 0.05). Therefore, it shows a correlation between the severity of sleep apnea syndrome and the severity and disability of patients with cerebral infarction through the NIHSS and mRankin scales. The mechanism of that combination can be explained as follows:

First, apnea and the resulting persistent O_2_ deficiency in patients with OSAS causes increased sympathetic tone and endothelial dysfunction. Vascular remodeling and vasoconstriction lead to cardiovascular complications, nocturnal hypertension, and other cardiovascular dysfunctions.

Second, oxidative stress which is produced by repeated hypoxemia and episodes of apnea leads to endothelial dysfunction and the rise of proinflammatory chemical mediators, such as Cyclooxygenase (COX-2), tumor necrosis factor-α (TNF-α), Interleukins and other pro-inflammatory chemical mediators. It easily leads to the initiation and progression of atherosclerotic plaque blood and insulin resistance.

Third, hypertension and arrhythmia, carotid intima-media thickness, and carotid atherosclerosis in the sleep apnea group are more common than in the normal group. Gonzaga found that the risk of hypertension was strongly associated with the potential severity of OSA after 4 years of follow-up (29). The blood flow in the middle cerebral artery remains unchanged due to the reaction of Angiotensin II, Noradrensine, Isoproterenol, and Bradykinin. Simultaneously, inhibition of plasminogen-1 and platelet activation leads to increased risk factors for vascular thrombosis.

## Conclusion

There is a significant proportion of cerebral infarction patients with OSA and this should be considered if the patient has symptoms such as loud snoring during sleep, excessive daytime sleepiness and large neck circumference, and high BMI. In addition, OSA is also a factor in predicting the severity of ischemic stroke patients.

## Data availability statement

The raw data supporting the conclusions of this article will be made available by the authors, without undue reservation.

## Ethics statement

The studies involving human participants were reviewed and approved by Ethics Committee of Hue University of Medicine and Pharmacy. The patients/participants provided their written informed consent to participate in this study.

## Author contributions

QD-M, TH-A, and SD-Q conceived the study and designed the study protocol. QD-M organized, performed the study investigations, and supported the recruitment of the patients. TH-A performed the statistical analyses. QD-M, TH-A, and NN-T-Y wrote the first draft of the manuscript. All authors have made substantial contributions, critically revised the manuscript for important intellectual content, and read and approved the final manuscript.
